# Chicago Gynæcological Society

**Published:** 1887-07

**Authors:** 

**Affiliations:** 2330 Indiana avenue


### CHICAGO GYNÆCOLOGICAL SOCIETY.

REGULAR MEETING, *Friday, April 15, 1887.*

I.—H. T. BYFORD.—1. Curettes.
  2. Round Ligaments from a Case of Alexander's operation.

II.—EARLE.—Cystic Sarcoma of Right Ovary.

III.—FENGER.—1. Proliferating Cystoma with Partial Intraligamentous Development.
  2. A new Colpoplastic Operation for Defect of the Vagina.
  3. Antisepsis in Abdominal Operations. Synopsis of a Series of Bacteriological Studies.

IV.—CALDWELL.—Enlarged Thyroid or Goitre a Cause of Transverse Presentation.

V.—TAIT.—Reply to Dr. M. Sänger.

THE PRESIDENT, CHARLES WARRINGTON EARLE, M. D., in the chair.

### I.—DR. H. T. BYFORD.

*1. Curettes.*

I want to show three curettes, which I have had made to answer a specific purpose not as well met by any other instrument. The ordinary method of removing the retained placenta in abortion is by the finger when possible. I have done this quite often, and have devised this largest curette to take the place of the finger. It is rounded in all directions on the end, has a spoon-shaped opening, is strong and yet flexible in the shank. It can be introduced without much dilatation of the os, and can be bent so as not to require the cervix to be drawn to the vaginal orifice. The shape gives it the advantage of being a sort of a feeler, incapable of doing great harm even under strong pressure. When used as a scoop it can easily be made to scratch off tissue that is not solid, that does not belong to the uterus, but it is not sharp enough to cut the uterine substance itself. The next, of medium size, is used as an ordinary uterine curette, which, although dull, is very strong, and capable of removing softened or degenerated mucous membrane. The firmness and thickness of this curette makes it different in action from the dull wire curette, which cannot be used with such force, or if used with force would be more apt to bruise the tissues. This smallest size is both for diagnosis and uterine curettement, and has the same characteristics; it requires scarcely any dilatation of the uterus, and will not cut healthy uterine tissue. I have left it unpolished on the end for the purpose of making it answer better as a palpator.

DR. JAGGARD: I do not think Dr. Byford's curettes differ from Mundé's in any essential particular.

DR. BYFORD: One advantage is that, being thicker and rounder on the end, it is not as capable of doing harm. Mine is scooped out in the shape of a spoon, and catches hold of particles better; I think it is more like Mundé's than any other. I have left the end unpolished for the purpose of a better palpation of soft substances. It is nickle-plated, although not polished on the end. I think the smaller one can be used with considerable success in feeling. I have used it at the office for the diagnosis between malignant growths and interstitial fibroids. By the amount of pressure we can use we get a knowledge of the bleeding surface, whether friable or solid. We can usually get some of the malignant growth or of the mucous polypi, but obtain nothing but blood from the intestitial fibroid.

2. *Round Ligaments From a Case of Alexander's Operation.*

I have here a specimen of the round ligaments, removed by the Alexander operation only a few hours ago. They are interesting on account of the extreme attenuation, which may account for some cases of failure in this operation. They are almost as thin as wrapping cords, and will give quite an idea of what some of these ligaments are after they are stripped of their fibrous coat and drawn out. In one operation which I performed there was just such a condition, and after a long, careful attempt to make them run, I broke one of them, and almost lost it. The other one I could not get entirely loose and only shortened it three-quarters of an inch. I met with one ligament twice the normal diameter, or over four times the diameter of this one; the opposite ligament in that case was about as thin as this one.

I have performed Alexander's operation six times.

II.—The President.

*Cystic Sarcoma of Right Ovary.*

I have an abdominal tumor to exhibit. As the history of the growth of the tumor is interesting—as also an operation for hernia which was performed upon the patient—it gives me great pleasure to read a letter from Dr. J. W. LaGrange, of Marion, Iowa, in whose charge she was for some years. He writes, " I have attended Mrs. D——since 1882. During that time, she has had several attacks of nausea, vomiting and intense pain in the bowels and stomach, these symptoms usually subsiding in thirty-six to forty-eight hours. In August, 1884, I was called to see her and found her suffering from the usual symptoms of gastric catarrh, but the malady did not yield to the usual treatment and on the third or fourth day I found that she had an old hernia, that had come down, from the excessive vomiting and retching. The hernia was femoral and about the size of a small orange. Taxis failed to reduce it, even when the patient was under the influence of an anæsthetic. We advised an operation, but the patient refused, saying that by the use of warm applications it would go back itself as it had done on different occasions. The next day hot applications and taxis failed to reduce it, and the patient began to have stercoraceous vomiting. We urged the necessity of an operation, the danger of delay, but the patient would not give her consent. So the fifth and sixth days passed, during which the symptoms were even more alarming, but the patient would not yield to an operation. The seventh day the patient gave her consent, and while we were making preparations she had a fainting spell and the intestine slipped back. Then she exclaimed, ' I told you so.' She made an excellent recovery; a truss was fitted and she remained quite well up to November 21, 1884, when I was sent for again and found the hernia down. Taxis under the influence of an anaesthetic failed to reduce it; and hot applications were tried. Past experience made us certain of this fact that the gut on former occasions was not strangulated but incarcerated. So we delayed advising an operation until the fourth day, when the symptoms became so strongly marked that we again urged an operation, but the patient refused on the same grounds as before, and insisted that time and hot fomentations would cause it to go back. We waited, persisting in the local treatment, and every day urging the necessity of operating and the danger of delay, but were met by the old argument. The fourteenth day came and the patient was so weak and exhausted and the symptons were so alarming that she consented to the operation. After cutting through the skin and the fasciae, to our surprise the contents of the bowels began to pour out in large quantity. We experienced great difficulty in opening the hernial sac, the different coverings having become organized into one common mass, thickened and interspersed with blood vessels. When the gut was reached it was a small knuckle of intestine lying in the bottom of the thickened mass. It was quite black and slight pressure with the end of the finger caused it to slip back into the abdomen. To our surprise the patient rallied and in a few days was convalescing. Everything she ate or took into her stomach passed out of the artificial anus; and there was no discharge whatever of fecal matter from the natural channel. The wound around the artificial opening healed kindly and the patient gradually gained strength, and by December 1st was able to sit up. December 25th she had a movement of the bowels from the natural channel. The discharges of fecal matter from the artificial opening gradually grew less and the opening healed up entirely in one week from this time. The patient remained well until October 16, 1886, when she had typho-malarial fever. In my examination at this time, I discovered in the right inguinal region a hardness, tender on pressure, outlines well marked, and involving the tissues of the right inguinal region and extending over the hypogastric region to the median line. The patient stated that she noticed this hardness first about a month previous (September 15, 1886). She had not menstruated for two months; but previous to this time had always been regular and the flow seemed to be natural. Examination of the womb revealed a large tumor, tense to the touch, involving the fundus, also a smaller one about the size of a hen's egg, attached to the left side of the organ. The mouth of the womb pointed to the right of the pelvis, and the probe passed about one inch into the cavity of the womb. There was a slight watery discharge during the early weeks, but no odor. At this time I diagnosed the case as a fibroid with cellulitis, and prescribed hot fomentations and local application of iodine. She did not complain of much pain. In about a month her typhoid symptoms subsided and the hardness in the right side grew less, but the tumor continued to increase in size and the outline in the hypogastric region was well marked. About this time I called in consultation Dr. H. Ristine, of Cedar Rapids, Iowa. After a careful investigation of the case and a careful consideration of the rapid growth of the tumor, and general appearance of the patient, we diagnosed the case as fibroid sarcoma and gave a very unfavorable prognosis. Under tonic and stimulating treatment with proper nourishment, her general health somewhat improved. Our treatment for the tumor was ergot by enema, with laudanum when needed to relieve the pain. Her chief distress was nausea and vomiting. During the latter part of my treatment there was a slight bloody discharge on two different occasions, which continued but a day or so, but there was no fetid smell. The growth did not increase as rapidly during the last month as when first noticed."

This lady came to Chicago about February 1, 1887, to consult Dr. W. H. Byford in regard to an operation. The malignant nature of the tumor being detected, it was not regarded justifiable to operate, and particular attention was paid to her nutrition and the relief of her pain, the details of which were left to me, as I lived in her immediate vicinity. She gradually failed, and finally died from inanition and exhaustion March 16th. Several convulsions took place during the last weeks of her life, probably from cerebral anæmia, and the apthæ, which completely filled her mouth, were most remarkable. The autopsy was performed by my brother, Dr. Frank Earle. After the abdomen was opened we looked carefully for the condition of the bowel as affected by the hernia. We found a fold of the intestine directly in front of the tumor, extending from the left obliquely to the hernial sac on the right, its omentum being intimately attached to the tumor. The lowest point of the intestinal loop was connected to the hernial sac by a small ligamentous band. The neck of the hernial sac was not at all closed, and the finger could easily be introduced to the depth of two inches. A part of the tumor had so grown that it pushed up the intestine, and probably kept it from descending into the sac. Posteriorly the growth was adherent to the bowels and imbeded in the pelvis. One of the ureters was largely dilated, probably from pressure of the tumor. Apart from its malignancy it would have been impossible to have removed it. Microscopically it was found to be in the main a cystic sarcoma, although there was a small fibroid at the fundus, which Drs. La Grange and Ristine had detected. The point of origin of the tumor was apparently the right ovary. The fundus was pressed to the left and anteflected.

To recapitulate: We had here a case that had been more or less an invalid for five years. Symptoms for four and a half years, referable to the stomach; repeated attacks of gastric catarrh with nausea, vomiting and pain; a hernia which had given great trouble; and finally, a rapidly growing tumor filling the abdomen, first noticed about six months previous to death, which seemed to be caused by inanition and exhaustion.

DISCUSSION.

DR. W. H. BYFORD: I have not heard the history of the case heretofore, as it has been given by the letter read this evening, but I arrived at the conclusion, when I saw her first, that it was a sarcomatous tumor, from the rapidity of its growth and the great emaciation of the patient, together with the discharges, which were a little different from a fibroid or any other kind of a tumor. I do not think it would be very disgraceful in me or anybody else, to acknowledge a good deal of ignorance about sarcomatous disease. Certainly our knowledge is not as definite and extensive upon this subject as upon several other malignant and benign diseases. I do not think that it would be worth while to enter upon the discussion of sarcoma at present.

DR. DANIEL T. NELSON: I have but a single suggestion, and am hardly prepared to make it satisfactorily: A sarcomatous growth like a cancer, is likely soon to become fused with adjacent tissues. But, if it can be discovered early enough and removed by enucleation, it seems to me there is a possible hope of permanent recovery. The removal of the whole of the disease, as in any malignant disease, is the thing to be aimed at, and I believe there is a time in cancer and in sarcoma when it can all be removed. We do not generally find the patient at such a time, yet I believe that in the future our means of diagnosis will be so much improved, and our skill so much increased, that we will be able to diagnose these cases much earlier, and remove the growth when the patient can be cured, and it is to urge the careful study of the diagnosis in these cases, and the question of early operation when they are diagnosed, that I have risen. It seems to me that in these two diseases, especially sarcoma, an early diagnosis is of importance, and if an operation is ever to be done it should be done early.

DR. C. T. PARKES: I do not think I shall have anything to say on the question of sarcomatous tumors. In the history of this case there is one rather interesting circumstance mentioned; that is, the recovery after the occurrence of sloughing of intestine from strangulated hernia. It is outside of the question of sarcoma, but goes to confirm my experience, that cases of strangulated hernia that have been allowed to remain for some time, with ensuing sloughing of the intestines, are not always fatal, nor do they always require subsequent operative procedures. I have seen two cases of strangulated hernia that were supposed to be abscesses, but when the parts were operated upon, and the abscess cavity exposed, at the bottom of it were found sloughing intestines, and both of these cases, artificial anus in each, did well and have recovered. The restoration was apparently complete, and up to the present time they have suffered no difficulty from the condition.

---

III.—Dr. Christian Fenger.

1.—*Proliferating Cystoma with Partial Intraligamentous Development.*

In addition to my remarks on parovarian cysts, at a former meeting of this society, I wish to present a specimen of a proliferating cystoma with partial intraligamentous or retroperitoneal development. It was taken from a woman 48 years old, operated upon March 22, 1887, in the Emergency Hospital. The photographs I pass around will show that the tumor was of enormous size. The abdomen measured at the umbilicus 132 centimeters in circumference, and from the xiphoid process to the symphysis pubis 63 centimeters: When, after tapping, the cyst was partially brought out of the wound, I found a very peculiar formation attached to the side of the cyst near the abdominal end of the enlarged Fallopian tube. As will be seen in the specimen passed around, it consists of a long, narrow cylindrical sac, 21 centimetres long and 4 centimetres in diameter. Its wall is almost transparent and half filled with a clear fluid. I have not been able to find described anywhere in the literature a similar appendix to any ovarian cyst. It seems to me likely to a reopened and dilated Morgagnian hydatid, the remnant of the terminal portion of Müller's duct. The retroperitoneal portion of the cystoma was enucleated as usual, leaving the large wound cavity with insufficient peritoneal covering. In this cavity Mikulicz's drain was used with a glass tube through its center. The last of the drain was removed on the 18th day, and the wound finally closed shortly afterward.

DISCUSSION.

Dr. W. H. Byford: I would like to make a few remarks on the subject of this specimen, particularly with reference to the manner of treating parovarian tumors. I suppose there are very few instances of parovarian tumors in which they become pedunculated. We do infrequently meet with instances where there is a fair pedicle, but they are exceptions to the rule, which is that they rise from within the broad ligament and often carry up with them a layer of fibrous tissue. And instead of being pedunculated, they commonly lie in a cup-shaped excavation in the broad ligament, extending down into the ligament some distance. There is therefore generally no way of getting rid of them except by enucleation. As was stated by Dr. Fenger at our last meeting, the general subject of enucleation was probably taught to Americans, at least, by Dr. Miner, o Buffalo. But at the time Dr. Miner wrote upon the subject we did not possess the same knowledge of the difference between broad ligament tumors and ovarian tumors that we now have. At that time we frequently confounded ovarian tumors with tumors of the broad ligament, and Dr. Miner did not confine himself to the consideration of this sort of tumor, but believed he was always enucleating a cystoma of the ovary when he removed a cyst from the abdominal cavity. We are now disposed to believe that most if not all his cases (he did not enucleate many) belonged to the variety of parovarian cysts. It is with the view of pointing out a few things in connection with the enucleation that I am now speaking. In practicing enucleation, as we have done heretofore, we make every effort, without much method, to get rid of the fibrous covering by tearing it off, without any special reference to the exact manner in which it is done. I have met with a few of these tumors in the last three or four years which have caused me to think differently on this subject, and made me to look more narrowly into the matter of enucleating them. About four years ago I was called upon to operate in a case of this kind in which there was considerable inflammation as well as a very thick coat of fibrous substance brought up with the upper layer of the broad ligament, enveloping the lower third of the tumor quite closely. In operating I found that by careful manipulation I could get out the cyst proper from the capsule without tearing down the side of it. The manipulation consisted in pushing the fingers down deep between the capsule and the cyst until they went below the tumor on all sides and in the center, when I found that I could get the sac entirely out, and in that instance succeeded in removing it without at all tearing down the sides of the enveloping capsule. I could then bring the edges of this cut-shaped stump to the external wound and stitch them to it. By means of a drainage tube in this cavity passing though the external wound I was able to drain off the fluid, while the whole raw surface of the hollow stump was excluded from the paritoneal cavity. I have done that operation myself now three times, and have seen it performed twice. Two weeks ago last Sunday I saw an operation at the Wonan's Hospital, performed by Dr. Henry T. Byford, for removal of one of these broad ligament tumors. After emptying the sac with the trocar, so that it was relaxed, he lifted it up as high as he could with Nélaton's forceps, then took the knife and lightly cut through the fibrous covering until he could get his fingers through it, when it was enucleated as above described. The inside of this cup-shaped pedicle or stump was entirely excluded from the peritoneal cavity, its edges were brought up to and included in the external wound and then a drainage tube introduced. This method of operating is always satisfactory, as, by making a perfectly close cup, the sides do not permit of the blood or serum getting into the peritoneal cavity. In two cases we found that no discharge came through the tube, hence it is probably not always necessary to drain. A remarkable thing in connection with the operations is that there is very little bleeding. In Dr. H. T. Byford's case the patient is convalescent, almost ready to go home; she lost less than four ounces of blood in the whole operation. There was no ligature placed upon any of the vessels. Dr. Fenger spoke of the operation being somewhat dangerous on account of the intestines. In this case the cæcum and the vermiform process were plainly visible upon the side of the tumor, but the fingers hulled out the capsule upon which they lay and left them perfectly free. I am convinced that these tumors can be cured as a general rule much more easily than by enucleation. I have not tried the process upon large tumors, but in small ones I make an incision through the abdominal walls, evacuate the tumor and leave a drainage tube in the sac. In four instances I have seen them cured in this way. These cysts are so simple in structure and have so little vitality that it is only necessary to drain them. Even tapping or aspirating them is sometimes sufficient. No dangers have arisen during the convalescence, and I am in hopes that the treatment of parovarian tumors on further experience will be very much simplified. I fully believe that evacuation and drainage of large parovarian cysts would be quite as successful as in the smaller ones.

---

3.—*Antisepsis in Abdominal Operations; Synopsis of a Series of Bacteriological Studies.*

(Preliminary Note, by Christian Fenger and Bayard Holmes.)

[NOTE.—This paper appears in another part of this issue as an original contribution.—*Editor.*]

---

IV.—DR. CHARLES CALDWELL.

*Enlarged Thyroid or Goitre a Cause of Transverse Presentation.*

June 1st, 1884, Mr. G—— summoned me to attend his wife. On the way to his house he informed me that she was in labor; that a midwife was in attendance and had been for twenty-four hours; that his wife did not seem to be making any headway, but was becoming weak and exhausted, and the midwife was frightened, not knowing what was the cause of the delay or what to do.

Her condition upon my entrance was truly frightful. She was in the midst of a labor pain, pulling the midwife's hands, straining every muscle, her whole face cyanotic, and her eyes protruding as if they would escape from their sockets. Exhausted she fell back gasping for breath.

It required but a single glance to perceive that death threatened this poor woman from two sources, *i. e.*, asphyxia and rupture of the uterus.

Bimanual examination revealed the fœtus in a transverse presentation. The os was fully dilated and the membranes had ruptured. The mother had not felt the movements of the fœtus for several hours and its heart sounds could not be heard.

Podalic version was performed without delay, by Braxton-Hicks' method. The feet were brought down, and during the next labor pain the second stage was completed. The third stage consumed but a few minutes and the uterus contracted well. The fœtus was large, but showed no signs of life. The heart's action had probably ceased several hours before.

After visiting my patient for three succeeding days, during which time her pulse and temperature remained normal, I saw no more of her, but her husband, whom I met the following week, informed me that she got up the fourth day and attended to her usual household duties.

Oct. 16, 1885, I was called a second time to attend Mrs. G—— in labor, and as in the first instance, she had called a midwife, also as before, she had been under her tender care, or rather at her tender mercy, for twenty-four hours. Again the fœtus was in a transverse presentation with the right arm and prolapsed cord protruding from the vulvar orifice, for the membranes had ruptured. The cord was pulseless. The hand and arm were easily pushed up over the face and above the brim of the pelvis.

By combined manipulation, with the right hand in the vagina and the left over the abdomen, cephalic version was performed. The head was brought down into the first position and held there until by a strong labor pain it became engaged, when forceps were applied at the superior strait and the fœtus extracted during the next pain.

This fœtus was an unusually large one, and I regret it was never weighed. Dr. Jaggard, who utilized it for the cause of obstetric science, says it must have weighed nearly or quite 20 lbs. Her puerperium was normal as before, which means she left her bed the fourth day.

February 18, 1887, Mr. G——, for the third, and, I hope, the last time, summoned me and said his wife needed my services again; that this time they had decided not to call a midwife first, but to send for me at once and give me a better opportunity to save the child's life. Examamination revealed a third case of transverse presentation. Os was fully dilated, but membranes unruptured. Through the membrane, by vagina, could be felt both the feet and hands. The head was to the right and breech to the left. Fœtus was in dorso-posterior position. The placenta could be easily felt through the abdominal walls, and was attached to the anterior wall of the uterus, near the fundus. By external manipulation, I attempted to perform cephalic version before the membranes ruptured. The amniotic fluid was small in quantity, and I failed. Deciding upon podalic version, the membranes were ruptured, and a fœtus weighing twelve pounds was delivered in a few minutes, but not without considerable muscular exertion on the mother's part as well as on my own.

As I brought down the feet with the right hand and pushed up the head with the left, the abdomen of the fœtus rotated anteriorly. As soon as the body was delivered, I clasped the thorax of the fœtus with the left hand to prevent inspiration and asphyxia. The arms were then brought down, and with the assistance of the nurse, who raised the child's body up over the mother's abdomen, I seized the fœtus by the occiput and delivered the after-coming head without forceps. The child gasped a few times, and then began to cry, to the delight of its mother. Placenta was soon expelled and the uterus contracted firmly and well.

Puerperium as in the two preceding cases.

The patient who furnishes me with my theme was born at Königsberg, Germany, in 1852; was married in 1873, and is the mother of ten children. The oldest four were born in Germany—the others in America, during the last nine years. Her first seven confinements were normal, and she was attended by a midwife. Seven years ago the left lobe of the thyroid gland began to enlarge during gestation, but caused neither pain nor dyspnœa. Three years ago, during her eighth pregnancy, the right lobe began to enlarge, and increased in size very rapidly, producing both pain and dyspnœa, compelling her to take a semi-recumbent position at night instead of a horizontal one. During the last three months of gestation she sat bolstered up in bed at night. This posture, which she was obliged to take whenever she sat down, during the day as well as the night, produced a continued pressure on the fundus of the uterus, changing its long axis from a vertical to an oblique or transverse direction. Of course, the long axis of the fœtus must coincide with that of the uterus, and the continued pressure on the breech or head, according to the end of the fœtal ovoid at the fundus, would force it into an oblique or transverse position, and also throw the lower end of the fœtus out of the pelvis and above its brim. The same position of the mother is maintained during labor as during the last three months of pregnancy, hence the fœtus remains in the same oblique or transverse position, and the lower end of the fœtal ovoid cannot descend below the pelvic brim.

It seems to me the above facts explain perfectly why the fœtus has been found in a transverse position, the three last confinements and never at a previous one.

In examining the obstetrical treatises at my command, I find no mention of enlarged thyroid as a cause of this presentation, but that it certainly is in this case I have not the least shadow of a doubt. The causes usually given are: Immature fœtus, contraction of pelvic brim, laxity of uterine muscles, excessive liquor amnii, twin pregnancies, etc., all of which can be excluded in this case.

The case has been quite interesting to me, for it is rare in my obstetric practice to meet with cases of transverse presentation, and I have *never* before seen it occur three times in succession in the same patient. I saw my patient last week, and find by measurement of the tumor at the largest point, that it has diminished more than an inch in its circumference in seven weeks, and that she can now sleep in bed with two pillows under her head. Dyspnœa is not alarming until the seventh month of pregnancy, but from that time until confinement it is excessive. This is hardly the place to discuss what mode of procedure should be followed in her case for her relief; and yet, as she is but thirty-five years old, it is very probable that she may become pregnant again, and possibly lose her life, if nothing is done to remove or at least reduce the size of the tumor, until it shall cease to be a source of such alarming dyspnœs.

I should be glad to hear the opinions of any of the Fellows who have had experience with this class of glandular tumors. From my own experience I should not be in favor of total extirpation, for I once saw a woman cross the river before the operation was completed.

Ligation of the superior and inferior thyroid arteries, partial resection, injections or electrolysis seem to offer better results, and are certainly much less dangerous.

DISCUSSION.

DR. D. T. NELSON: I have had one patient that this case reminds me of, in which no shoulder or other irregular presentation was the cause of difficult labor, but a high promontory (and it seems to me with the experience I have had with abnormal presentations that a high promontory is a very common cause of mal-position), complicated by an enlarged thyroid, not to the extreme extent of the case reported, but such as to give the patient a great deal of inconvenience in the last months of pregnancy, and especially at the time of delivery. I have delivered her with instruments several times. The treatment I have adopted in her case for the enlarged thyroid, and with seeming benefit, has been the free use of ergot in the intervals between pregnancies. The gland will diminish greatly in size, at least in the intervals between pregnancies, as the pressure is taken off of the circulation. When she followed the treatment with some degree of faithfulness (she would not use the remedy with persistency), it seems to me that in two periods, when she did fairly well, continuing it for about two months, there was a more marked improvement than at any other time. I would suggest this to the Doctor, as I know of nothing else that is more likely to serve him, before he decides upon a surgical operation. In the acute enlargement of the thyroid, occurring in both sexes at puberty, though most frequently in the female, I have given ergot with excellent results, continuing it several months.

DR. C. T. PARKES: I would suggest, as to the treatment of this thyroid gland, that among the many methods I have seen adopted, the injection of a 5 per cent. solution of carbolic acid sometimes brings about a very rapid improvement, and decrease of the enlarged gland. An injection with a hypodermic syringe once or twice a week is not followed by any unpleasant reaction.

---

V.—MR. LAWSON TAIT. *Reply to Dr. M. Sænger*, 51, 7, The Crescent;

BIRMINGHAM, England, March 4, 1887.

*To Charles Warrington Earle, M.D., President of the Chicago Gynæcological Society*:

SIR: I have just read Dr. Sænger's reply to a letter which I sent to the Chicago Gynæcological Society, concerning Dr. Sænger's divisions and subdivisions of pyosalpinx.

Dr. Sænger seems a little wroth that I should have regarded these subdivisions as absurd, but I still continue to do so, and I am perfectly certain that the position which I take concerning them will be admitted before long to be a just and accurate one. However antagonistic that attitude may be to Dr. Sænger, it does not justify him in using the language which he does.

He says, for instance: "Lawson Tait's practical results are so good that they have raised his conceit to so high a degree that on pathological questions he assumes a certain infallibility which vents itself in numerous sallies and attacks upon others."

I trust this is not true. My numerous sallies and attacks are not induced by conceit on my own part, but by misrepresentations on the part of others. Dr. Sænger himself forms an example of that misrepresentation. He says that "Lawson Tait has asserted that menstruation does not depend upon the ovaries but upon the tubes." This is a direct misrepresentation. It is not I who have asserted that menstruation does not depend upon the ovaries, but Ritchie asserted it and proved it before I was born. Reeves Jackson, of Chicago, has sustained the proof, so have Balfour, Melassey and a dozen more, but the proof does not yet seem to have penetrated the German mind. I have sustained the proof by abundant clinical examples, and some of my clinical work has indicated that the tubes have a large influence upon the production of the phenomena of menstruation. This is all I have had to do with the matter, and Dr. Sænger has no right to be so ignorant as to indicate any misrepresentation of this kind when he assumes the position of a dogmatic instructor.

Dr. Sænger says that my statements are reckless, and he expresses this concerning the statement of Cæsarian Section having a mortality of 99.9 per cent. I asserted that that *was* the mortality of Cæsarian Section. Dr. Sænger misrepresents me again in saying that I said it *is still* the mortality. I suppose that he must think I am alluding to some modification of his own which may have been in a small group of cases more or less successful. I alluded to nothing of the kind. I alluded to the past history through generations of this fatal operation, and every one, in this country at least, knows that not anything approaching one in a hundred cases recovers. This is not a reckless statement of mine, it is an opinion which has been displayed by every contribution to the literature of obstetrics during the last one hundred years in England. It induced Simpson to make his celebrated joke that "Cæsarian Section was not successful unless it was done by a cow."

Dr. Sænger asks me if I have been taught nothing by the researches of Semmelweis and Lister, Pasteur and Koch. I do not know the first of the four. In Lister I have seen a humiliating example of an eminent man of science warped by a delusion and obliged to maintain absolute silence in face of adverse criticism. The researches of Pasteur and Koch have long since, in the opinion of many here been blown to the winds. The germ theory of disease and the details of practice which have been based upon it are such as suit the metaphysical tendencies of the German mind, and afford elaborate opportunities for subtle definitions and distinctions, as exemplified in Dr. Sænger's paper upon pyo-salpinx. These speculations, however, like others of their kind, are based upon the fallacy that the theories and facts of the process of decomposition are made to do duty for the theories and facts of the disease. But those of us who have clinical materials to work upon, and care to observe and carefully record clinical facts, know well that living tissue does not follow the same processes which infusions of dead meat do in the laboratory of the biologist.

If the cause of the salpingitis be the germ, which is so satisfactory to the mind of Dr. Sænger, why is it that the disease is confined absolutely to the tubes in the great bulk of instances? Why do we not have acute suppurating metritis? Why does not the inflammatory action extend all over the peritonenum and to every viscus in the body? What is there in the fimbriated extremities which has such a powerful antiseptic influence in arresting the progress of this all-powerful germ? All these absurd crotchets have fortunately had but a short hold on the English understanding, and their tenure is rapidly approaching an end.

I have neither time nor inclination to re-enter upon the controversy of the last ten years, in which I have taken an active share, in showing my reasons for the disbelief alike of the germ theory and practices based upon it. But I shall speedily have an opportunity of addressing myself to this question, and I shall take up the text of Dr. Sænger's utterances upon pyo-salpinx.

To a teacher of the germ theory such divisions of disease as Dr. Sænger seems to believe in may afford a large amount of comfort, but to the practical surgeon they are useless and delusive, whilst neither in clinical material nor in pathological research have yet found support.

I can not regard the man who seriously believes in the production of tubercular disease by the presence of a bacillus or its location in the tube by accidental contact with the vulva as being more rational or reasonable than the man who believes in the bacillus of an earthquake. The whole blunder of the germ theory of disease has been the mistake of association for causation.

Dr. Sænger asks me what disease before the introduction of Listerism killed thousands of patients who had received wounds or who had been operated upon. I say that nothing more completely answers his question than the one word DIRT.

Dr. Sænger again asks me what is puerperal fever? We, in England, are beginning to understand that a whole lot of different things are mixed up under that term, and at present I am not prepared to say anything more definite about it than that our answer to this important question has been greatly hindered by this mischievous germ theory. Dr. Sænger says that this has been demonstrated and the other theory has been proved. We do not accept any one of these assertions. I am, etc.,

LAWSON TAIT.

P. S.—I have just seen the conclusion of Dr. Sænger's article, and regard as amusingly inconsistent the advice given with the example set by Dr. Sænger in the style of language he adopts. His statement that neither before nor after the operation do I concern myself with the nature of the disease treated, is absolutely untrue. Further, the statement that I admit that in every fifth case I make an error in diagnosis, is a gross misrepresentation of the facts. What I have said is that ubal disease can be easily diagnosed, but that differential diagnosis of the contents of the tubes whether they be serum, pus or blood, can not be made with certainty in more than four out of five cases.

If Dr. Sænger can do better than this, there must be one of three special explanations :

I. He must be a heaven-inspired gynæcologist.

II. Tubal disease must be very different in Germany from what it is in England.

III. Or he is evolving the whole thing from his inner consciousness without any more real knowledge than his countryman had of the celebrated camel.

In conclusion let me say that when Dr. Sænger tells me that in Germany they have long since ceased to regard as serious my theoretical utterances, which pretend to be scientific, he is manufacturing the whole by a process of imagination. I have made no pretense to be scientific, and have advanced no theories. I have adhered rigidly to facts. It is Dr. Sænger who has invented the theories and mischievously stuck my name on them.

L. T.

W. W. JAGGARD, M.D. Editor,
2330 Indiana avenue.

